# Influence of reproductive experience on the antidepressant-like effect of estradiol and prolame in middle-aged rats

**DOI:** 10.3389/fnbeh.2026.1937892

**Published:** 2026-10-22

**Authors:** Olivia Tania Hernández-Hernández, José Jaime Herrera-Pérez, Stefani Michell González-Longoria, Lucía Martínez-Mota

**Affiliations:** 1 Unidad de Investigación en Reproducción Humana, Secretaría de Ciencia, Humanidades, Tecnología e Innovación-Instituto Nacional de Perinatología-Facultad de Química, Universidad Nacional Autónoma de México, Fisiología y Desarrollo Celular, Ciudad de México, Mexico; 2 Laboratorio de Farmacología Conductual, Instituto Nacional de Psiquiatría Ramón de la Fuente Muñiz, Dirección de Investigaciones Biomédicas en Salud Mental, Ciudad de México, Mexico

**Keywords:** antidepressant effects, estradiol, females, middle-aged, prolame, reproductive experience

## Abstract

**Introduction:**

Reproductive experience may affect the antidepressant action of estrogen replacement therapy (ERT). In the most used animal model of menopause, ovariectomized young adult rats, prolame produces estrogenic and antidepressant-like effects, making it a potential alternative to conventional ERT. A previous study indicated that aging cancels out the antidepressant-like effect of estradiol and reduces the window of antidepressant action of prolame, limiting it to only 3 weeks after ovariectomy. However, the aged females (middle-aged) used in that study were retired breeders, a factor that could explain their antidepressant response to both estrogens. Thus, the present study aimed to determine how reproductive experience influences the antidepressant-like effects of estradiol (30 μg/kg) and prolame (60 μg/kg) in middle-aged Wistar rats.

**Methods:**

The antidepressant-like effect of both estrogens was evaluated 3 weeks after ovariectomy in nulliparous and multiparous middle-aged rats using the forced swim test. We also assessed the effect of estrogens on uterine weight.

**Results:**

Our results suggest that reproductive experience confers stress resilience. Prolame, but not estradiol, produced an antidepressant-like effect in middle-aged female rats, both multiparous and nulliparous. Treatment with estradiol and prolame increased uterine weight similarly; however, the uterus of nulliparous rats showed greater sensitivity to estrogens.

**Conclusion:**

Taken together, the results suggest that prolame might be a viable therapeutic alternative for perimenopausal or postmenopausal women to alleviate depressive symptoms, and a candidate for ERT.

## 1 Introduction

Natural menopause occurs after 12 consecutive months without menstruation, and it typically occurs between the ages of 45 and 55 years. At this stage, women exit the reproductive phase due to a significant decline in ovulatory function and the cessation of menstruation (Harlow et al., 2012). On the other hand, perimenopause is defined as the phase in middle age during which a woman transitions from a reproductive to a non-reproductive period. The transition to menopause is characterized by fluctuations in estrogen levels, which are associated with the deterioration of ovarian function linked to the cessation of reproductive life. This hormonal imbalance generates a range of symptoms, including hot flushes, insomnia, irritability, diminished libido, cognitive alterations and depression (Soares, 2013; Uddenberg et al., 2024).

Depression is a mental disorder characterized by symptoms such as sadness, anhedonia, parasomnias, feelings of guilt and impaired concentration. This affective disorder represents the primary cause of disability among women on a global scale, resulting in a substantial impairment of their normal functioning and quality of life (DSM-V, 2022). The prevalence of depression in women is twice that of men (Kessler, 2003; Marcus et al., 2005), which has been linked to psychosocial and biological etiological factors, such as stress associated with gender roles, inequality in access to health services, and sex-dependent variations in gonadal hormones. In this sense, specific periods associated with fluctuations in estrogen levels throughout the female reproductive cycle represent “windows of vulnerability” for the onset of a new episode of depression or its recurrence (Sassarini, 2016). This vulnerability is particularly relevant for middle-aged women, who may manifest depressive symptoms or even the recurrence of major depressive disorder (MDD) (Brown et al., 2024) requiring treatment to improve their mental health (Bromberger and Kravitz, 2011; Joffe et al., 2020).

Perimenopause and menopause have historically been managed with estrogen replacement therapy (ERT) to alleviate the most prominent symptoms, including hot flushes, insomnia, and genitourinary alterations (Soares, 2023). Perimenopause and menopause-related depression lack a specific pharmacological treatment capable of alleviating the constellation of symptoms observed in middle-aged women; however, ERT has been proposed as a potential treatment for depression during these periods (Rasgon et al., 2002), with reports of the efficacy of ERT mainly under the schedule of transdermal administration in small samples of women (Xiang et al., 2024). Interestingly, ERT treatment predicts improved mood in menopausal women with MDD, associated with increased estradiol levels, while a secondary analysis showed that this improvement was observed in perimenopausal women but not in postmenopausal women (Joffe et al., 2020).

The use of ERT to treat depression may be limited due to diverse factors such as the type of estrogen and the female endocrine condition, the timing of estrogen replacement initiation after the estrogenic decline, either surgical or natural, and aging (Walf and Frye, 2005; Estrada-Camarena et al., 2010). Consideration of all these factors may be essential for an effective ERT (Hernández-Hernández et al., 2022). Furthermore, the potential for adverse effects of these hormones, which include increased risk of breast or endometrial cancer, as well as thromboembolic events (Marjoribanks et al., 2017) restricts the benefits of the currently used ERTs. Therefore, it is necessary to find alternative estrogenic molecules that balance benefits and risks to treat depression and improve women's mental health during menopause. In this regard, the synthetic 17β-aminoestrogen prolame (17β-(3-hydroxy-1-propylamino)−1,3,5(10)-estratrien-3-ol) is an estradiol analog in which the C17 position of the steroid nucleus is substituted with an aminoalcohol side chain (NH(CH_2_))_3_OH (Fernandez et al., 1985). Like estradiol, studies in rodents have shown that prolame produces effects on the uterus and gonadotropin release, albeit with less potency than the natural steroid (Jaimez et al., 2000). Unlike estradiol, which has procoagulant effects, prolame has anticoagulant effects, potentially making it beneficial for ERT in menopausal women at increased risk of thromboembolic events (Lemini et al., 2005).

Animal models can help elucidate the mechanisms underlying disease and changes during human menopause, helping address health problems at this physiological stage in women (Herrera-Pérez et al., 2024). In animal models, the decrease in estrogen levels that occurs during menopause can be simulated by bilateral ovariectomy. The forced swimming test (FST) is a model in which acute stress induces behavioral changes, such as behavioral despair in rodents, reflected in immobility behavior (Porsolt et al., 1978; Detke et al., 1995). Ovariectomy increases immobility behavior in the FST; this can be reduced by administering estradiol to physiological levels (Walf and Frye, 2005). Like estradiol, prolame administration produces antidepressant-like effects in ovariectomized rats subjected to the forced swimming test (FST) (Lemini et al., 2016; Hernández-Hernández et al., 2022), an extensively used laboratory test for screening the effectiveness of antidepressant-like drugs (Porsolt et al., 1978; Detke et al., 1995). Interestingly, middle-aged or older female rats are a better model than young adult rats for studying perimenopause and menopause. (Herrera-Pérez et al., 2024). Using this model, a previous study showed that estradiol and prolame reduced immobility in young adult rats 3 and 8 weeks after ovariectomy, whereas only prolame had an effect in middle-aged rats 3 weeks post-ovariectomy (Hernández-Hernández et al., 2022). Thus, the antidepressant-like effects of estradiol and prolame seem to be influenced by age and the timing of ERT initiation following post-ovariectomy, but other factors should be identified, since the middle-aged rats used in this study were retired breeders.

In this regard, Walf and Frye (2008) proposed that reproductive experiences (copulation, gestation, parturition, and lactation) should be considered when evaluating the effects of estradiol on affective behavior. This consideration stems from the fact that the reproductive experience can trigger changes in hormonal secretion in humans and rats, which permanently alter the mother’s brain (Bridges and Byrnes, 2006; de Lange et al., 2020).

In a study conducted by Barha and Galea (2011)they determined the acute effects of 17β-estradiol, 17α-estradiol, and estrone on hippocampal cell proliferation in middle-aged ovariectomized female rats, and whether these effects depended on prior reproductive experience. They found that all three estrogens increased hippocampal cell proliferation in middle-aged multiparous rats but had no effect in middle-aged nulliparous rats. Therefore, prior reproductive experience may cause the aging brain to respond more intensely to estrogens in adulthood (Barha and Galea, 2011). Altogether, physiological changes associated with parity and aging may interact to modulate the response to ERT commonly used in menopause.

Although some studies suggest that reproductive experience influences the central nervous system’s (CNS) response to estradiol, it has not been investigated whether such experience modulates the antidepressant actions of estrogens, particularly in middle-aged females who may need ERT to improve mood and avoid MDD recurrence. Therefore, this study aimed to determine whether reproductive experience influences the antidepressant-like effects of estradiol and prolame in ovariectomized middle-aged rats using the forced swimming test. Additionally, we analyzed the trophic effects of estradiol and prolame on the uterus of these rats to assess their safety.

## 2 Materials and methods

### 2.1 Animals

Female Wistar rats (15 months old, middle-aged, weighing approximately 300–500 g) were obtained from the vivarium of the Instituto Nacional de Psiquiatría Ramón de la Fuente Muñiz (INPRFM). Female rats have a life expectancy of 2 to 3 years, exhibiting irregular reproductive cycles in middle-aged (8–12 months) and becoming acyclic at 17–21 months (Brann and Mahesh, 2005). Of the 48 rats used, 24 were nulliparous (had never given birth), and 24 were multiparous (retired breeders who had given birth to 3–4 litters). Rats were housed in groups of 4 per cage under an inverted light/dark cycle (12 h/12 h, lights on at 9:00 pm), at a controlled temperature (23 ± 2 °C), with *ad libitum* access to food (Purina rat chow) and water. The INPRFM Ethics Committee approved the experimental protocol (No. CEI/C/047/2017). Animal care was in accordance with Mexican Official Norm NOM-062-ZOO-1999. We made every possible effort to reduce the number of rats used and minimize pain and discomfort.

### 2.2 Experimental design

To evaluate the influence of reproductive experience on the antidepressant-like effects of estradiol and prolame, independent groups of nulliparous and multiparous ovariectomized middle-aged rats received one of the following treatments: estradiol (30 μg/kg), prolame (60 μg/kg), or corn oil as the vehicle (1 mL/kg). All treatments were evaluated for 3 weeks after surgery (*n* = 8 per group). Treatments were administered subcutaneously once daily for 3 days: the first injection was given immediately after the pre-test, the second 24 h after the pre-test, and the last 4 h before the FST test session (Section 2.5.1). Moreover, to control the confounding effects of treatments that could influence the interpretation of the FST results, rats were evaluated in the OFT 1 h before the test session. After finishing the experiment, rats of each group were euthanized by decapitation, and their uteri were removed (Figure 1).

**Figure 1:**
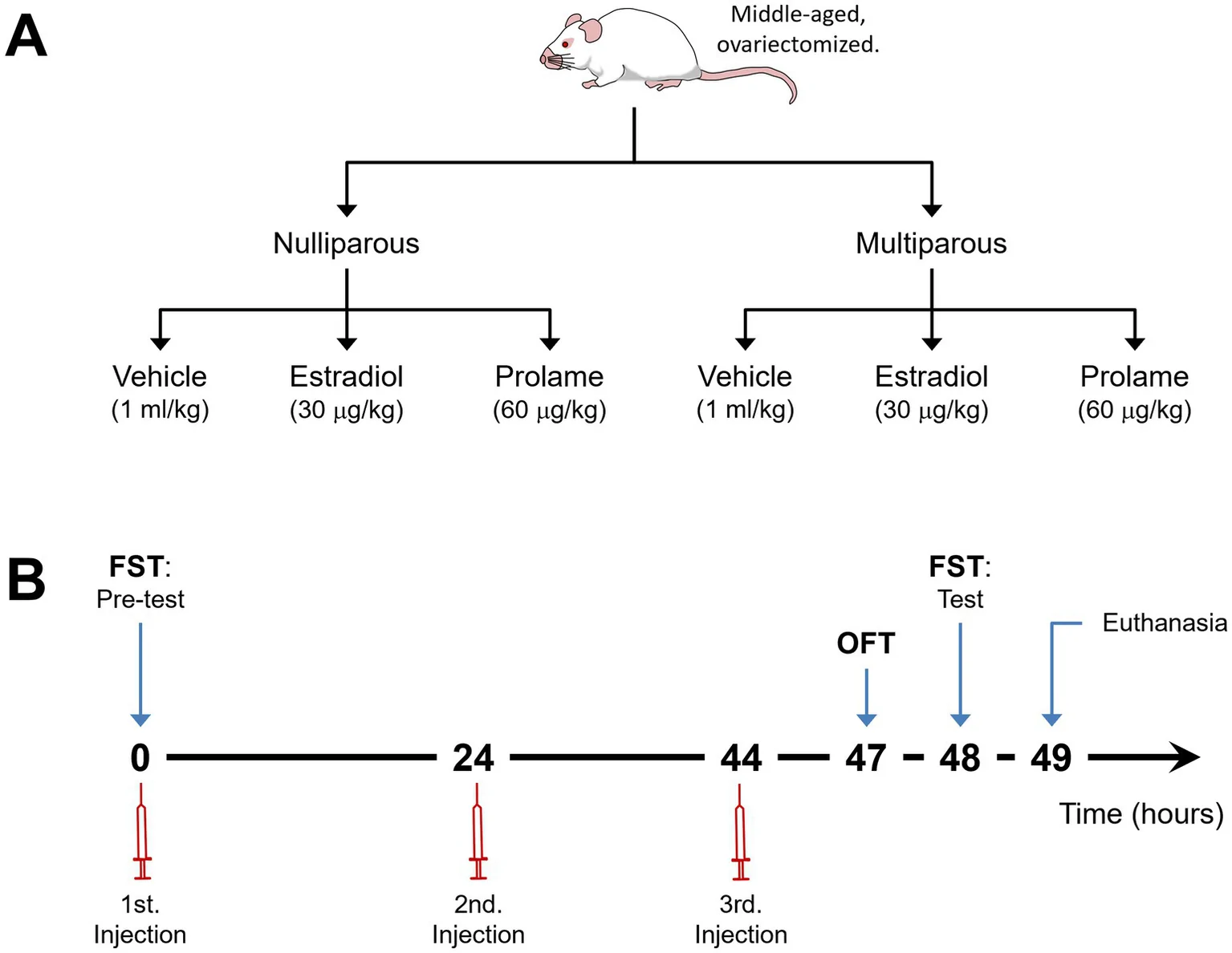
**(A)** Experimental design. Independent groups of ovariectomized nulliparous and multiparous middle-aged rats received one of the following treatments: estradiol (30 μg/kg), prolame (60 μg/kg), or corn oil as the vehicle (1 mL/kg). We evaluated all treatments for 3 weeks after surgery (n = 88 per group). **(B)** Time line for behavioral test and drug administration. Treatments were administered subcutaneously once daily for 3 days: the first injection was given immediately after the pre-test, the second 24 h after the pre-test, and the last 4 h before the Test session. Open field test (OFT) was conducted 1 h before the Test session. After the forced swimming test (FST), rats were euthanized and their uteri were removed.

### 2.3 Ovariectomy

Bilateral ovariectomy was performed under aseptic conditions in middle-aged rats anesthetized with 2,2,2-tribromoethanol (Fluka, 200 mg/kg, i.p) dissolved in 0.9% saline solution containing 8% ethanol. An incision was made in the rat’s ventral midline skin and muscle, and the ovaries were exposed. The ovarian arteries were bilaterally ligated to avoid bleeding, and the ovaries were removed. The skin and muscle were sutured and cleaned with an antiseptic solution (chloride benzalkonium, Antibenzil, Farmacéuticos Altamirano de México, S.A. de C.V.). Afterward, rats were returned to their home cages to recover for 3 weeks before treatment. For 3 days of postoperative care, rats received the antibiotic enrofloxacin (1 mg/mL in drinking water) and the analgesic ketorolac (0.3 mg/mL in drinking water).

### 2.4 Drugs

Prolame 17β-(3-hydroxy-1-propylamino)−1,3,5(10)-estratrien-3-ol was synthesized from estrone. The detailed process of prolame synthesis has been described elsewhere (Fernandez et al., 1985). Prolame was subjected to chemical purity testing using analytical (MP, TLC) and spectroscopic (IR, NMR, MS) methods and compared with authentication samples. Estradiol (3,17β-dihydroxy-1,3,5(10)-estratriene) was purchased from Sigma-Aldrich (St. Louis, MO, USA). Estradiol and prolame were dissolved in corn oil (Mazola, México) before their administration. These solutions were freshly prepared before experiments.

The doses of estradiol and prolame, as well as the treatment regimens, were selected based on their antidepressant-like effects in ovariectomized young and middle-aged female rats. We previously demonstrated that estradiol at 30 μg/kg and prolame at 60 μg/kg reduced immobility behavior and increased uterine weight at 3 and 8 weeks post-ovariectomy in young rats; this effect was also observed in middle-aged rats, although only for prolame administered 3 weeks after ovariectomy (Hernández-Hernández et al., 2022) whether in a subacute (3 days) or chronic regimen (26 days) (Lemini et al., 2013, 2016). Consequently, we used the 3-day treatment regimen.

The administration of these doses of estrogens (estradiol at 30 μg/kg and prolame at 60 μg/kg) to ovariectomized rats stimulates sexual behavior, reduces anxiety-like behaviors, and reverses deficits in spatial learning and memory caused by the loss of ovarian hormones—desired effects in ERT(Lemini and Canchola, 2009; Nissen et al., 2012). Under this regime of estrogen treatment, a previous study indicated that the estradiol serum levels in rats treated with estradiol were 387.28 ± 50.89 pg/mL for young rats, and 451.18 ± 84.70 pg/mL for middle-aged rats; meanwhile, after prolame administration, serum estradiol levels were 40.26 ± 2.88 and 100.15 ± 16.42 pg/mL in young adults and middle-aged rats, respectively. Estradiol levels under both treatments were higher than in the vehicle-treated groups (young adults: 16.65 ± 1.40 pg/mL and middle-aged: 43.59 ± 9.80 pg/mL) (Hernández-Hernández et al., 2022). Estradiol levels during the proestrus phase of the estrous cycle in young adult rats are 88 ± 2 pg/mL, representing the highest peak hormone concentration (Butcher et al., 1974). These levels are close to those obtained with prolame treatment in middle-aged rats and are supraphysiological with estradiol treatment. Conversely, the normal range of estradiol levels in women during the phases of the menstrual cycle is as follows: for the follicular phase, between 22.2 and 233 pg/mL; for the ovulation period, between 41 and 398 pg/mL; during the luteal phase: 22 to 341 pg/mL; and in postmenopausal women not receiving ERT: 0 to 30 pg/mL (Glynne et al., 2025).

These data suggest that the estrogen doses used achieve estradiol concentrations within the physiological range and should mimic those used in ERT. Regarding the estrogen doses used in this study and whether these conditions could be applied to ERT, a study conducted on perimenopausal and postmenopausal women who used transdermal estradiol—either as a patch, gel, or spray—for ≥3 months (estradiol dose of 0.5 to 4 mg/day, in gel form), reported a serum estradiol concentration of 355.26 pmol/L. It was also reported that the optimal plasma estradiol concentration for relieving menopausal symptoms and preventing bone loss is 60–150 pg/mL. It was determined that, at a concentration of 60 pg/mL, hot flashes are relieved in 50% of women and bone resorption is prevented; 100% relief from hot flashes and bone accretion occur when levels approach 100 pg/mL (Steingold et al., 1985; Lignieres, 1996; Glynne et al., 2025). It is important to note that ERT doses administered to women should be individualized based on the women’s menopausal symptoms or mood.

### 2.5 Behavioral tests

#### 2.5.1 Forced swimming test (FST)

The FST is based on observations of rodents facing an unavoidable stressful situation and how they employ coping strategies that can be classified as active or passive behaviors. The modified FST includes a pre-test session designed to induce despair, as evidenced by a gradual increase in immobility and a decrease in active behaviors, typically accompanied by elevated serum corticosterone levels (Detke et al., 1997). Active behaviors (climbing and swimming) predominate during initial exposure to the FST, but over time they are typically replaced by passive behavior (immobility) (Molendijk and de Kloet, 2022). The main advantage of the FST is its predictive validity, which refers to the ability of drug or sex hormones to promote active behavior and reduce immobility during the FST; that is, its potential to predict antidepressant efficacy. Immobility in the FST has been associated with an impaired stress response and difficulties adapting to change, although many studies describe it as depressive-like behavior (Commons et al., 2017) FST is also sensitive to variables such as age and the post-ovariectomy endocrine milieu, revealing variations in active or passive behaviors (Olivares-Nazario et al., 2016; Puga-Olguín et al., 2019).

Rats were randomly assigned to receive the vehicle, estradiol, or prolame before the pre-test session. In a previous study in our laboratory with young and adult female rats, we analyzed immobility, swimming, and climbing behaviors during the 15 min pre-test, divided into three 5-minute intervals. In the first interval, from 0 to 5 min, a similar number of counts was observed for immobility, swimming, and climbing behaviors; subsequently, an increase in immobility and a decrease in the active behaviors of swimming and climbing were observed in the 5- to 10-min and 10- to 15-min intervals, with the greatest increase in immobility (50 counts) occurring in the last interval (Martínez-Mota et al., 2011). In the test session, rats that do not receive hormonal treatment show high immobility counts, but when they are administered an antidepressant drug or a hormone with antidepressant effects, immobility counts decrease significantly (Martínez-Mota et al., 2011).

Rats were evaluated during the dark phase using the modified FST proposed by Detke et al., (1997). Rats were placed in a glass cylinder (46 cm high × 20 cm diameter) containing a 30 cm column of water at 23 °C–25 °C. Two swimming sessions were conducted. The first was a pre-test session (15 min) used to reveal the antidepressant action of estrogens in the second (test) session (5 min), carried out 48 h later. After each swim session, we removed the rats, dried them, and placed them in heated cages for 30 min before returning them to their home cages. We conducted test sessions between 12:00 and 15:00 and video-recorded them for subsequent scoring. Because the influence of the pre-test on the test session lasts at least 21 days and the effects of antidepressant drugs can be observed during this period (Detke et al., 1997) we decided to evaluate active or passive behaviors 48 h after the pre-test session.

An experimenter blind to the experimental condition used a time sampling technique to score every 5 s the presence of three behaviors: (1) immobility—floating without struggling, and making only those movements necessary to keep the head above the water; (2) swimming active motions around the cylinder, more than required to keep the head above water; and (3) climbing - rats making active movements with their forepaws in and out of the water, usually directed against the wall (Detke et al., 1995). Drugs that reduce immobility scores were considered to produce an antidepressant-like effect, which may be accompanied by increases in swimming, climbing, or both behaviors (Detke et al., 1995). Thus, in a 5 min test, a total of 60 counts, including immobility, swimming, or climbing, were scored. The frequency of each behavior was expressed as the mean ± standard error of the mean (SEM).

#### 2.5.2 Open field test

A locomotor test was conducted 5 min before the test swimming session to rule out confounding hormonal actions on the behavioral changes observed in the FST. The rats were evaluated for 5 min in an automated system (PanLab, Spain) consisting of a plexiglass cage (45 × 35 × 45 cm) with two infrared sensors mounted on the cage walls (2.5 and 10.5 cm from the cage base) coupled to the IR LE8811 software. The system detected and recorded all locomotor activity (movement from one place to another) in the rats during the 5 min test, including spontaneous motor activity and exploratory behavior (Carter and Shieh, 2015). Locomotor activity was expressed as mean ± SEM.

### 2.6 Uterine weight

After the behavioral tests, the rats were euthanized; the uterus was removed, thoroughly cleaned of fat, and weighed. The weight of the uterus was related to body weight to obtain the uterine index (UI), using the following formula:

Uterine index(UI)=uterine weight(mg)/body weight(g).

To assess differences in the uterus's response to treatment between nulliparous and multiparous rats, the normalized uterine index (NUI) for each reproductive status was calculated. In this calculation, the uterine index was expressed as a percentage relative to the mean uterine index of the group treated with vehicle, using the following formula:

Normalized uterine index(NUI)=[UItx/MUIveh]×100

where UItx refers to the individual uterine index for any treatment, and MUIveh refers to the mean uterine index for the vehicle-treated group.

### 2.7 Statistical analysis

We analyzed the results using a two-way analysis of variance (ANOVA), considering reproductive experience and estrogen treatment as independent variables. Tukey's test was used as a post-hoc test. A *p*-value < 0.05 was considered statistically significant.

## 3 Results

### 3.1 Forced swimming test

Present results suggest that reproductive experience reduces immobility behavior and increases both active behaviors, indicating that multiparity improves resilience to stress in middle-aged rats. It is also clear that in middle-aged rats, prolame and estradiol had different behavioral effects, with prolame being the only one that effectively affected their behavior in the FST.

Two-way ANOVA indicated main effects of reproductive experience and estrogen treatment on the behaviors evaluated in the forced swim test; no interaction between these factors was found in any case. For immobility, reproductive experience had a main effect (*F*_1,48_ = 28.267, *p* < 0.001), with multiparous rats showing less immobility than nulliparous rats (*p* < 0.001). Estrogen treatment (*F*_2,48_ = 23.511, *p* < 0.001) also influenced this behavior; post-hoc analysis indicated that prolame treatment reduced immobility compared with vehicle-treated rats (*p* < 0.001), whereas estradiol treatment did not affect this behavior (*p* = 0.967). Similarly, reproductive experience affected swimming behavior (*F*_1,48_ = 5.837, *p* = 0.02), with counts higher in multiparous rats than in their nulliparous counterparts (*p* = 0.02); furthermore, swimming was affected by estrogen treatment (*F*_2,48_ = 21.771, *p* < 0.001), and in this case, only prolame increased the behavior compared to rats treated with the vehicle (*p* < 0.001). Regarding climbing behavior, differences were determined by reproductive experience (*F*_1,48_ = 23.771, *p* < 0.001) or estrogen treatment (*F*_2,48_ = 4.058, *p* = 0.024), with multiparous rats showing higher levels of the behavior compared to nulliparous rats (*p* < 0.001), and rats treated with estradiol showing less climbing behavior than rats treated with prolame (*p* = 0.018). The absence of interaction between reproductive experience and estrogen treatment indicates that the action of estrogens on behaviors did not depend on reproductive experience (Figure 2).

**Figure 2:**
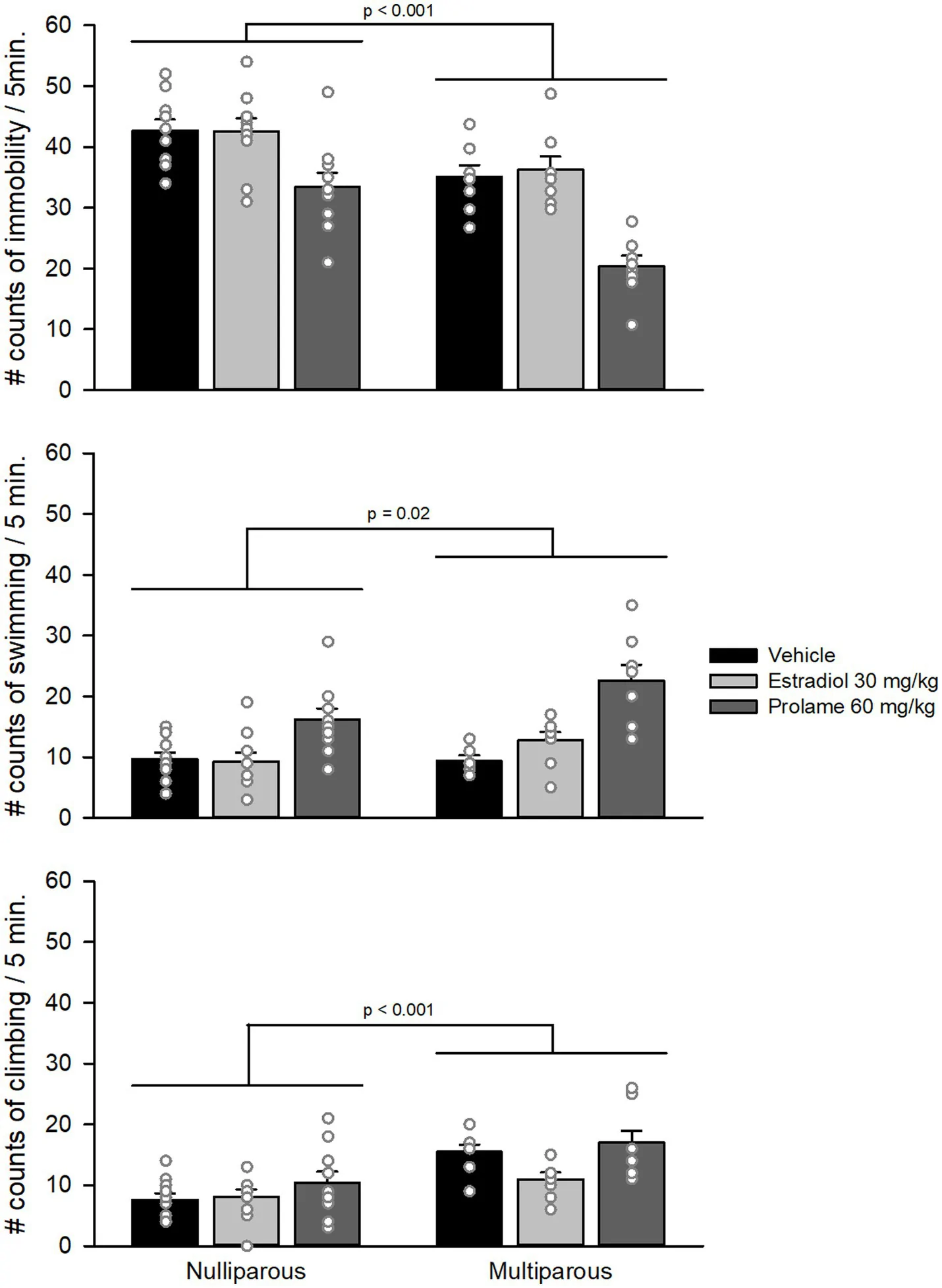
Influence of reproductive experience and hormone treatment on behaviors exhibited by middle-aged rats in the forced swimming test. Counts of immobility, swimming and climbing behaviors are shown. Results are expressed as mean ± SEM.

Together, these data indicate that prolame, but not estradiol, had an antidepressant effect in middle-aged rats, regardless of reproductive experience.

### 3.2 Open field test

Behavioral data in the forced swimming test reflect specific antidepressant-like actions, as suggested by the two-way ANOVA for locomotor activity, which indicated that this parameter was not affected by reproductive experience (*F*_1,48_ = 0.522, *p* = 0.473), treatment (*F*_2,48_ = 0.253, *p* = 0.778), or the interaction of both factors (*F*_2,48_ = 1.751, *p* = 0.185) (Table 1).

**Table 1:** Locomotor activity of middle-aged nulliparous and multiparous rats.

Reproductive experience	Vehicle	Estradiol	Prolame
		(30 μg/kg)	(60 μg/kg)
Nulliparous	2234.0 ± 173.4	2410.4 ± 239.5	2603.5 ± 231.2
Multiparous	2573.3 ± 336.5	2112.1 ± 116.4	2173.0 ± 124.1

Results are expressed as mean ± SEM.

### 3.3 Uterine weight

#### 3.3.1 Uterine index

The index results are shown in Figure 3A. Two-way ANOVA indicated a main treatment effect (*F*_2,48_ = 20.014, *p* < 0.001); differences were attributable to an increase in this parameter in rats treated with estradiol or prolame compared with the vehicle-treated group (*p* < 0.001 in both cases). This analysis did not indicate differences attributable to reproductive experience (*F*_1,48_ = 0.0294, *p* = 0.865) or by the interaction between both factors (*F*_2,48_ = 0.512, *p* = 0.602). Thus, the sensitivity of uterine index to estrogen treatment did not depend on reproductive status.

**Figure 3:**
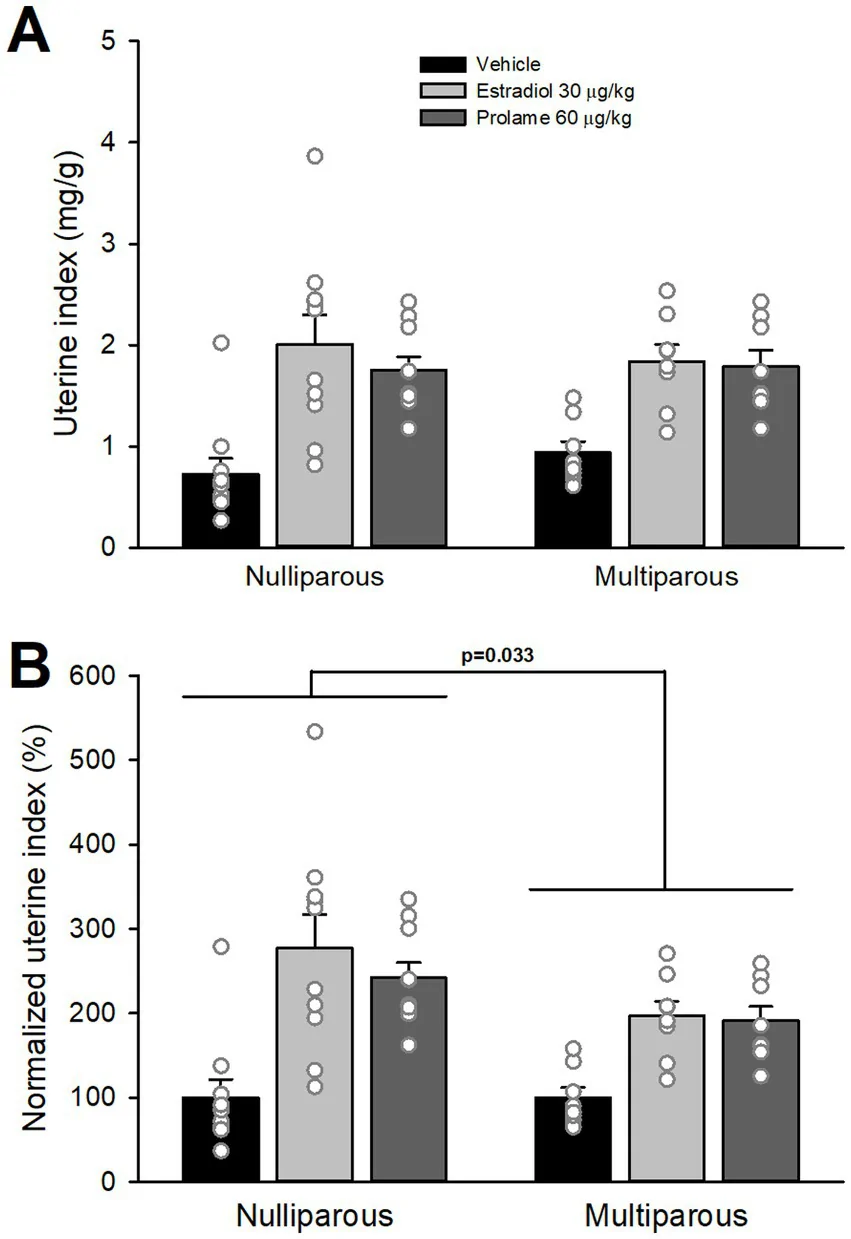
Effect of reproductive experience and hormone treatment on uterine weight of middle-aged rats. Uterine index **(A)** and normalized uterine index **(B)** are shown. Results are expressed as mean ± SEM.

These data suggest that, contrary to the antidepressant effect, both estrogens exert similar actions on uterine tissue in nulliparous or multiparous rats.

#### 3.3.2 Normalized uterine index

The results for the normalized uterine index are shown in Figure 3B. Two-way ANOVA for this index indicated differences attributable to reproductive experience (*F*_1,48_ = 4.801, *p* = 0.033) and treatment (*F*_2,48_ = 18.11, *p* < 0.001), but not to their interaction (*F*_2,48_ = 1.379, *p* = 0.262). Post-hoc analysis showed that the normalized uterine index was lower in multiparous than in nulliparous rats (*p* = 0.033); furthermore, this index increased in rats treated with estradiol or prolame (*p* < 0.001 in both cases).

Analysis of this parameter suggested that the uterus of nulliparous rats is more sensitive to estrogen than that of multiparous rats.

## 4 Discussion

During perimenopause, climacteric symptoms may be associated with the onset of a new or recurrent depressive episode, reflecting changes in ovarian hormone and neurotransmitter levels. In postmenopause, the response to certain antidepressants may depend on whether the patient also receives ERT (Wise et al., 2008). To achieve optimal results, treatments for depression should consider the woman’s reproductive stage and associated biological and endocrine factors.

In the present study, we determined whether reproductive experience influences the antidepressant-like effects of estradiol and prolame in ovariectomized middle-aged nulliparous or multiparous rats. We also analyzed the trophic effects of estradiol and prolame on uterine weight to assess their safety.

Prolame elicited antidepressant-like effects in ovariectomized middle-aged rats, regardless of reproductive experience; furthermore, it increased swimming behavior as assessed by the FST. In contrast, estradiol did not induce antidepressant actions in ovariectomized middle-aged rats, whether nulliparous or multiparous. Another relevant FST finding is that immobility behavior, indicative of behavioral despair, was lower in multiparous rats. Regarding the trophic effects of estradiol and prolame, it was demonstrated that both estrogens increase uterine weight in both nulliparous and multiparous rats. Furthermore, according to the normalized uterine index data, the uterine response to estrogen treatment in nulliparous female rats was higher than that in multiparous rats, suggesting that uterine tissue in nulliparous rats is more sensitive to estrogen. Additionally, estradiol and prolame did not affect the locomotor activity of nulliparous or multiparous rats, confirming that the antidepressant-like effect of estrogens is specific.

### 4.1 Influence of reproductive experience and treatment on the locomotor activity test

Some drugs can produce false positives in the FST by increasing locomotor activity, thereby facilitating swimming and climbing in the test (Porsolt et al., 1977). The locomotor activity test helps to exclude nonspecific antidepressant effects of drugs (Porsolt et al., 1978). The results indicate that estradiol and prolame doses administered to nulliparous and multiparous rats did not alter locomotor activity compared with vehicle-treated rats. Changes in reproductive status in females did not alter locomotor activity. The data suggest that neither reproductive experience nor treatments altered general locomotor activity; moreover, the effects of both estrogens observed in the FST are not related to locomotor activity but rather to their antidepressant activity.

### 4.2 Multiparous rats exhibit fewer immobility behaviors

Reproductive experience can have lasting effects on the maternal brain since significant changes occur in the immune, endocrine, and nervous systems during pregnancy and the postpartum period (Puri et al., 2023). Neuronal dynamics vary across stages of reproduction, from gestation to the postpartum period. Thus, the observed changes in the maternal brain could be associated with decreased hippocampal cell proliferation at mid-gestation and in the early postpartum period (8 and 30 days after partum) in primiparous rats, but this later normalized. Interestingly, an increase in hippocampal neurogenesis was reported in middle-aged multiparous rats compared with nulliparous rats (Barha et al., 2015). In both primiparous and multiparous rats, cell proliferation decreased notably during the early postpartum period, accompanied by a decline in cell survival. Moreover, brief exposure of pups to nulliparous rats significantly enhanced proliferation, whereas 22-day exposure to sensitized nulliparous rats increased cell survival. Collectively, these findings suggest that reproductive experience markedly influences hippocampal neurogenesis (Pawluski and Galea, 2007). Multiparous rats exhibit higher levels of brain-derived neurotrophic factor (BDNF) and synaptophysin, as well as more immature neurons, than nulliparous rats in middle age (Barha et al., 2015). The plasticity in maternal brains is associated with changes in affective and cognitive functions.

In this regard, a study of adult multiparous rats found that they spent more time in the open arms of the elevated plus maze test than nulliparous rats, suggesting that rats with reproductive experience exhibit less anxiety-like behavior (Walf and Frye, 2008). Thus, female rats that have experienced one or more pregnancies and one or more caregiving stages (primiparous and multiparous) show greater resilience to stress, less anxiety, and better memory skills than female rats that have never experienced motherhood (nulliparous) (Macbeth and Luine, 2010).

In the present study, multiparous rats exhibited less behavioral despair in the FST than nulliparous rats, evidencing that reproductive experience confers resilience in middle-aged female rats. Consistent with this idea, Pawluski et al. (2009) suggest that reproductive experience may generate resilience against depressive and anxiety-like behaviors in female rats. The study included primiparous and nulliparous female rats in several conditions: primiparous rats (with exposure to pups), primiparous rats without exposure to pups, nulliparous rats, and nulliparous rats sensitized by exposure to pups. Primiparous females not exposed to pups exhibited depressive-like behavior compared to sensitized nulliparous and primiparous females. It is suggested that maternal exposure to offspring produces effects like those of antidepressants. A positive association has been found between the time spent breastfeeding, kyphosis (arched-back nursing), and the percentage of time spent swimming, suggesting reduced depressive behavior among mothers who spent more time in kyphotic breastfeeding postures. Thus, exposure to offspring, lactation, and motherhood are important for maternal emotionality (Pawluski et al., 2009).

Our findings indicate that multiparous rats exhibit more swimming counts in the FST than nulliparous rats, suggesting that reproductive experience enhances swimming behavior. Consistent with these findings, parity in young female Sprague–Dawley rats has been documented to increase swimming, decrease the percentage of time spent immobile in the FST, and facilitate recovery following a stress-induced elevation in corticosterone levels (Workman et al., 2016). Antidepressants that act on the serotonergic system increase swimming behavior, whereas those that act on the noradrenergic system increase climbing in rats exposed to the FST (Detke et al., 1995). Estrada-Camarena et al., (2011) reported similar results, evaluating the effects of two common antidepressants, fluoxetine, a selective serotonin reuptake inhibitor, and desipramine, a norepinephrine reuptake inhibitor, in ovariectomized rats. These findings confirm that the serotonergic system modulates swimming and that the noradrenergic system modulates climbing behavior in females. They also suggest that reproductive experience promotes activation of serotonergic and noradrenergic pathways. Interestingly, and supporting this idea, successful maternal behavior in rodents depends on serotonergic neurotransmission, as a deficiency in serotonin synthesis negatively affects the quality of care and offspring survival (Lerch-Haner et al., 2008).

Taken together, these data suggest that reproductive experience reduces immobility behavior despair and increases climbing and swimming, indicating that multiparity enhances stress resilience in middle-aged rats.

### 4.3 Prolame, but not estradiol, induces antidepressant effects in middle-aged nulliparous and multiparous rats

This study determined that prolame exhibits an antidepressant effect in middle-aged rats three weeks following ovariectomy; this effect was observed to be consistent in both multiparous and nulliparous females. In both groups, estradiol did not demonstrate an antidepressant effect. The antidepressant profiles of estradiol and prolame align with those previously reported in middle-aged multiparous rats (Hernández-Hernández et al., 2022).

A study on middle-aged ovariectomized female rats examined whether reproductive experience influences the effects of acute treatments with 17β-estradiol, 17α-estradiol, or estrone on hippocampal cell proliferation. All estrogens significantly increased cell proliferation in the hippocampus of multiparous rats, but not in nulliparous rats, suggesting that reproductive experience may facilitate the brain responding to estrogens (Barha and Galea, 2011). In addition, Galea et al. (2018) investigated the effects of Premarin (a clinically used estrone-based estrogen) on early spatial learning and memory in ovariectomized nulliparous and multiparous rats; six months later, the rats were administered Premarin. Treatment with Premarin reduced the density of cells expressing doublecortin in the ventral dentate gyrus of primiparous rats and in the dorsal dentate gyrus of nulliparous rats compared to controls. These findings indicate that parity has lasting effects on the brain and behavior of middle-aged rats and that reproductive experience modifies the effect of Premarin (Galea et al., 2018). These results emphasize the importance of considering reproductive experience when implementing hormone therapy.

A common characteristic of these investigations and the present study is the subchronic nature of estradiol treatment, suggesting that chronic administration may be required for estradiol to elicit an antidepressant response in ovariectomized middle-aged rats. However, this idea is discarded since Lemini et al. (2024) reported that in ovariectomized middle-aged rats, prolonged treatment (26 days) with estradiol did not induce antidepressant or anxiolytic effects; in addition, these authors also showed that, in contrast to estradiol, chronic treatment with prolame induced antidepressant and anxiolytic effects in these rats, further supporting the results of the current study.

### 4.4 Reproductive experience alters uterine weight and sensitivity to estrogen

This study examined the trophic effects of estradiol and prolame on the uterus as a safety criterion for ERT. Both nulliparous and multiparous rats showed a similar uterine index, which increased after treatment with either estrogen. Normalization of this index demonstrated that the uterus of nulliparous rats was more sensitive to estrogen treatment than that of multiparous rats. These findings align with those reported by other researchers (Bridges and Byrnes, 2006; Walf and Frye, 2008), indicating that reproductive experience modulates ER expression in the uterus. Consequently, the observed differences in uterine sensitivity to estrogens between nulliparous and multiparous rats are likely attributable to neuroendocrine changes associated with reproductive experience (Walf and Frye, 2009).

The increase in the uterine index induced by treatment with estradiol and prolame is consistent with the findings reported by Lemini et al. (2005) for young adult female rats and mice, which were administered a vehicle (3 mL/kg), prolame, butolame, pentolame, or estradiol at doses ranging from 0.01 to 100 μg/kg for 3 days. In this study, uterine weight increased with estradiol and prolame treatment. The increase in uterine weight is primarily due to ERα activation, which stimulates cell proliferation and consequently increases uterine weight.

## 5 Conclusion

Our results suggest that, although reproductive experience confers resilience to stress, the antidepressant-like actions of estrogens used here are not related to reproductive experience. Notably, prolame, but not estradiol, produced an antidepressant-like effect in middle-aged female rats, whether multiparous or nulliparous, which supports the idea that prolame has important central actions linked to estradiol-independent mechanisms. Treatment with estradiol and prolame increased uterine weight to a similar extent; however, the uterus of nulliparous rats exhibited greater sensitivity to estrogens. Altogether, these results suggest prolame as a candidate for ERT and a viable therapeutic alternative for individuals to alleviate the symptoms of depression.

## Data Availability

The original contributions presented in the study are included in the article/supplementary material, further inquiries can be directed to the corresponding author.

## References

[ref1] Barha C. K. Galea L. A. M. (2011). Motherhood alters the cellular response to estrogens in the hippocampus later in life. Neurobiol. Aging 32, 2091–2095. doi: 10.1016/j.neurobiolaging.2009.12.004, 20034703.

[ref2] Barha C. K. Lieblich S. E. Chow C. Galea L. A. M. (2015). Multiparity-induced enhancement of hippocampal neurogenesis and spatial memory depends on ovarian hormone status in middle age. Neurobiol. Aging 36, 2391–2405. doi: 10.1016/j.neurobiolaging.2015.04.007, 25998101.

[ref3] Brann D. W. Mahesh V. B. (2005). The aging reproductive neuroendocrine axis. Steroids 70, 273–283. doi: 10.1016/j.steroids.2004.12.008 16080236

[ref4] Bridges R. Byrnes E. (2006). Reproductive experience reduces circulating 17 beta estradiol and prolactin levels during proestrus and alters estrogen sensitivity in female rats. Endocrinology 147, 2575–2582. doi: 10.1210/en.2005-0917, 16484327.

[ref5] Bromberger J. T. Kravitz H. M. (2011). Mood and menopause: findings from the Study of Women’s Health Across the Nation (SWAN) over 10 years. Obstet. Gynecol. Clin. North Am. 38, 609–625. doi: 10.1016/j.ogc.2011.05.011 PMC3197240 21961723

[ref6] Brown L. Hunter M. S. Chen R. Crandall C. J. Gordon J. L. Mishra G. D. . (2024). Promoting good mental health over the menopause transition. Lancet 403, 969–983. doi: 10.1016/S0140-6736(23)02801-5, 38458216.

[ref7] Butcher R. L. Collins W. E. Fugo N. W. (1974). Plasma concentration of LH, FSH, prolactin, progesterone and estradiol-17β throughout the 4-day estrous cycle of the rat. Endocrinology 94, 1704–1708. doi: 10.1210/endo-94-6-1704, 4857496.

[ref8] Carter M. Shieh J. (2015). “"Animal behavior",” In: Guide to Research Techniques in Neuroscience 2nd (ed). San Diego, CA: Academic Press. p. 39–71. doi: 10.1016/978-0-12-800511-8.00002-2

[ref9] Commons K. G. Cholanians A. B. Babb J. A. Ehlinger D. G. (2017). The rodent forced swim test measures stress-coping strategy, not depression-like behavior. ACS Chem. Neurosci. 8, 955–960. doi: 10.1021/acschemneuro.7b00042, 28287253. PMC5518600

[ref10] de Lange A. G. Barth C. Kaufmann T. Maximov I. I. van der Meer D. Agartz I. . (2020). Women’s brain aging: effects of sex-hormone exposure, pregnancies, and genetic risk for Alzheimer’s disease. Hum. Brain Mapp. 41, 5141–5150. doi: 10.1002/hbm.25180, 32856754. PMC7670641

[ref11] Detke M. J. Johnson J. Lucki I. (1997). Acute and chronic antidepressant drug treatment in the rat forced swimming test model of depression. Exp. Clin. Psychopharmacol. 5, 107–112. doi: 10.1037/1064-1297.5.2.107 9234045

[ref12] Detke M. J. Rickels M. Lucki I. (1995). Active behaviors in the rat forced swimming test differentially produced by serotonergic and noradrenergic antidepressants. Psychopharmacology (Berl) 121, 66–72. doi: 10.1007/BF02245592, 8539342.

[ref13] Estrada-Camarena E. López-Rubalcava C. Hernández-Aragón A. Mejía-Mauries S. Picazo O. (2011). Long-term ovariectomy modulates the antidepressant-like action of estrogens, but not of antidepressants. J. Psychopharmacol. 25, 1365–1377. doi: 10.1177/0269881111408456, 21890587.

[ref14] Estrada-Camarena E. López-Rubalcava C. Vega-Rivera N. Récamier-Carballo S. Fernández-Guasti A. (2010). Antidepressant effects of estrogens : a basic approximation. Behav. Pharmacol. 21, 451–464. doi: 10.1097/FBP.0b013e32833db7e9 20700047

[ref15] Fernandez J. M. Rubio-Arroyo M. F. Soriano-García M. Toscano R. A. Pérez-César M. C. (1985). Synthesis and molecular structure of prolame, N-(3-hydroxy-1,3,5(10)- estratrien-17β-yl)-3-hydroxypropylamine; an amino-estrogen with prolonged anticoagulant and brief estrogenic effects. Steroids 45, 151–157. doi: 10.1016/0039-128x(85)90044-3 3841424

[ref16] Galea L. A. M. Roes M. M. Dimech C. J. Chow C. Mahmoud R. Lieblich S. E. . (2018). Premarin has opposing effects on spatial learning, neural activation, and serum cytokine levels in middle-aged female rats depending on reproductive history. Neurobiol. Aging 70, 291–307. doi: 10.1016/j.neurobiolaging.2018.06.030 30056312

[ref17] Glynne S. Reisel D. Kamal A. Neville A. McColl L. Lewis R. . (2025). The range and variation in serum estradiol concentration in perimenopausal and postmenopausal women treated with transdermal estradiol in a real-world setting: a cross-sectional study. Menopause 32, 103–111. doi: 10.1097/GME.0000000000002459, 39689249. PMC12147738

[ref18] Harlow S. D. Gass M. Hall J. E. Lobo R. Maki P. Rebar R. W. . (2012). Executive summary of the stages of reproductive aging workshop + 10: addressing the unfinished agenda of staging reproductive aging. J. Clin. Endocrinol. Metab. 97, 1159–1168. doi: 10.1210/jc.2011-3362, 22344196. PMC3319184

[ref19] Hernández-Hernández O. T. Herrera-Pérez J. J. Chavira R. Lemini C. Martínez-Mota L. (2022). Effects of post-ovariectomy time frame and age on the antidepressant-like actions of estradiol and Prolame in female rats. Horm. Behav. 141:105154. doi: 10.1016/j.yhbeh.2022.105154, 35306314.

[ref20] Herrera-Pérez J. J. Hernández-Hernández O. T. Flores-Ramos M. Cueto-Escobedo J. Rodríguez-Landa J. F. Martínez-Mota L. (2024). The intersection between menopause and depression: overview of research using animal models. Front. Psychiatry 15:1408878. doi: 10.3389/fpsyt.2024.1408878, 39081530. PMC11287658

[ref21] Jaimez R. Cooney A. Jackson K. Lemus A. E. Lemini C. García R. . (2000). In vivo estrogen bioactivities and in vitro estrogen receptor binding and transcriptional activities of anticoagulant synthetic 17 b -aminoestrogens 73, 59–66. doi: 10.1016/s0960-0760(00)00053-4 10822025

[ref22] Joffe H. de Wit A. Coborn J. Crawford S. Freeman M. Wiley A. . (2020). Impact of estradiol variability and progesterone on mood in perimenopausal women with depressive symptoms. J. Clin. Endocrinol. Metab. 105, e642–e650. doi: 10.1210/clinem/dgz181, 31693131. PMC7075107

[ref23] Kessler R. C. (2003). Epidemiology of women and depression. J. Affect. Disord. 74, 5–13. doi: 10.1016/S0165-0327(02)00426-3 12646294

[ref24] Lemini C. Canchola E. (2009). Effects of 17 β -aminoestrogens on the sexual behavior of female rats. Physiol. Behav. 96, 662–666. doi: 10.1016/j.physbeh.2009.01.003 19385021

[ref25] Lemini C. Cruz-López B. Martínez-Mota L. (2013). Participation of estrogen receptors in the antidepressant-like effect of prolame on the forced swimming test. Pharmacol. Biochem. Behav. 103, 659–665. doi: 10.1016/j.pbb.2012.11.004, 23159542.

[ref26] Lemini C. Franco Y. Avila M. E. Jaimez R. (2005). Estrogenic effects of 17β-aminoestrogens assessed in uteri of rats and mice. Eur. J. Pharmacol. 510, 235–239. doi: 10.1016/j.ejphar.2005.01.028, 15763248.

[ref27] Lemini C. García-Albor E. Cruz-López B. Matamoros-Trejo G. Márquez-Baltazar S. Herrera-Pérez J. J. . (2024). Prolame produces anxiolytic- and antidepressant-like effects in middle-aged female rats with less uterotrophic effects than 17β-estradiol. Eur. J. Pharmacol. 969:176454. doi: 10.1016/j.ejphar.2024.176454, 38417607.

[ref28] Lemini C. García-Albor E. Cruz-López B. Matamoros-Trejo G. Martínez-Mota L. (2016). Differential effect of the 17 β -aminoestrogens prolame, butolame and pentolame in anxiety and depression models in rats. Prog. Neuropsychopharmacol. Biol. Psychiatry 64, 102–108. doi: 10.1016/j.pnpbp.2015.07.013, 26239795.

[ref29] Lerch-Haner J. K. Frierson D. Crawford L. K. Beck S. G. Deneris E. S. (2008). Serotonergic transcriptional programming determines maternal behavior and offspring survival. Nature Neurosci. 11, 1001–1003. doi: 10.1038/nn.2176, 19160496. PMC2679641

[ref30] Lignieres B. (1996). Hormone replacement therapy: clinical benefits and side-effects. Maturitas 23, S31–S36. doi: 10.1016/S0378-5122(96)90012-2 8865137

[ref31] Macbeth A. H. Luine V. N. (2010). Changes in anxiety and cognition due to reproductive experience: a review of data from rodent and human mothers. Neurosci. Biobehav. Rev. 34, 452–467. doi: 10.1016/j.neubiorev.2009.08.011, 19761791.

[ref32] Marcus S. M. Young E. A. Kerber K. B. Kornstein S. Farabaugh A. H. Mitchell J. . (2005). Gender differences in depression: findings from the STAR*D study. J. Affect. Disord. 87, 141–150. doi: 10.1016/j.jad.2004.09.008 15982748

[ref33] Marjoribanks J. Farquhar C. Roberts H. Lethaby A. Lee J. (2017). Long-term hormone therapy for perimenopausal and postmenopausal women. Cochrane Database Syst. Rev. 2017, 1–135. doi: 10.1002/14651858.CD004143.pub5 PMC6465148 28093732

[ref34] Martínez-Mota L. Ulloa R.-E. Herrera-Pérez J. Chavira R. Fernández-Guasti A. (2011). Sex and age differences in the impact of the forced swimming test on the levels of steroid hormones. Physiol. Behav. 104, 900–905. doi: 10.1016/j.physbeh.2011.05.027, 21658399.

[ref35] Molendijk M. L. de Kloet E. R. (2022). Forced swim stressor: trends in usage and mechanistic consideration. Eur. J. Neurosci. 55, 2813–2831. doi: 10.1111/ejn.15139, 33548153. PMC9291081

[ref36] Nissen I. Estrada F. S. Nava-Kopp A. T. Irles C. Fernandez-G J. M. Govezensky T. . (2012). Prolame ameliorates anxiety and spatial learning and memory impairment induced by ovariectomy in rats. Physiol. Behav. 106, 278–284. doi: 10.1016/j.physbeh.2012.02.019, 22369853.

[ref37] Olivares-Nazario M. Fernández-Guasti A. Martínez-Mota L. (2016). Age-related changes in the antidepressant-like effect of desipramine and fluoxetine in the rat forced-swim test. Behav. Pharmacol. 27, 22–28. doi: 10.1097/FBP.0000000000000175, 26237710.

[ref38] Pawluski J. L. Galea L. A. M. (2007). Reproductive experience alters hippocampal neurogenesis during the postpartum period in the dam. Neuroscience 149, 53–67. doi: 10.1016/j.neuroscience.2007.07.031, 17869008.

[ref39] Pawluski J. Lieblich S. Galea L. (2009). Offspring-exposure reduces depressive-like behavior in the parturient female rat. Behav. Brain Res. 197, 55–61. doi: 10.1016/j.bbr.2008.08.001 18760310

[ref40] Porsolt R. D. Anton G. Blavet N. Jalfre M. (1978). Behavioral despair in rats: a new model sensitive to antidepressant treatments. Eur. J. Pharmacol. 47, 379–391. doi: 10.1016/0014-2999(78)90118-8 204499

[ref41] Porsolt R. D. Le Pichon M. Jalfre M. (1977). Depression: a new animal model sensitive to antidepressant treatments. Nature 266, 730–732. doi: 10.1038/266730a0 559941

[ref42] Puga-Olguín A. Rodríguez-Landa J. F. Rovirosa-Hernández M. de J. Germán-Ponciano L. J. Caba M. Meza E. . (2019). Long-term ovariectomy increases anxiety- and despair-like behaviors associated with lower Fos immunoreactivity in the lateral septal nucleus in rats. Behav. Brain Res. 360, 185–195. doi: 10.1016/j.bbr.2018.12.017, 30529405.

[ref43] Puri T. A. Richard J. E. Galea L. A. M. (2023). Beyond sex differences: short- and long-term effects of pregnancy on the brain. Trends Neurosci. 46, 459–471. doi: 10.1016/j.tins.2023.03.010, 37120339.

[ref44] Rasgon N. L. Altshuler L. L. Fairbanks L. A. Dunkin J. J. Davtyan C. Elman S. . (2002). Estrogen replacement therapy in the treatment of major depressive disorder in perimenopausal women. J. Clin. Psychiatry 63, 45–48. 11995778

[ref45] Sassarini D. J. (2016). Depression in midlife women. Maturitas 94, 149–154. doi: 10.1016/j.maturitas.2016.09.004 27823736

[ref46] Soares C. N. (2013). Depression in peri- and postmenopausal women: prevalence, pathophysiology and pharmacological management. Drugs Aging 30, 677–685. doi: 10.1007/s40266-013-0100-1, 23801148.

[ref47] Soares C. N. (2023). Menopause and mood. Psychiatr. Clin. N. Am. 46, 463–473. doi: 10.1016/j.psc.2023.04.004 37500244

[ref48] Steingold K. A. Laufer L. Chetkowski R. J. Defazio J. D. Matt D. W. Meldrum D. R. . (1985). Treatment of hot flashes with transdermal estradiol administration. J. Clin. Endocrinol. Metab. 61, 627–632. doi: 10.1210/jcem-61-4-627 3928674

[ref49] Uddenberg E. R. Safwan N. Saadedine M. Hurtado M. D. Faubion S. S. Shufelt C. L. (2024). Menopause transition and cardiovascular disease risk. Maturitas 185:107974. doi: 10.1016/j.maturitas.2024.107974 38555760

[ref50] Walf A. A. Frye C. A. (2005). Antianxiety and antidepressive behavior produced by physiological estradiol regimen may be modulated by hypothalamic – pituitary – adrenal axis activity. Neuropsychopharmacology 30, 1288–1301. doi: 10.1038/sj.npp.1300708, 15756306.

[ref51] Walf A. Frye C. (2008). Parity and estrogen-administration alter affective behavior of ovariectomized rats. Physiol. Behav. 93, 351–356. doi: 10.1016/j.physbeh.2007.09.002 PMC2754137 17936862

[ref52] Walf A. A. Frye C. A. (2009). Effects of two estradiol regimens on anxiety and depressive behaviors and trophic effects in peripheral tissues in a rodent model. Gend. Med. 6, 300–311. doi: 10.1016/j.genm.2009.04.004 PMC3616371 19467526

[ref53] Wise D. D. Felker A. Stahl S. M. (2008). Tailoring treatment of depression for women across the reproductive lifecycle: the importance of pregnancy, vasomotor symptoms, and other estrogen-related events in psychopharmacology. CNS Spectr. 13, 647–662. doi: 10.1017/S1092852900013742 18704021

[ref54] Workman J. L. Gobinath A. R. Kitay N. F. Chow C. Brummelte S. Galea L. A. M. (2016). Parity modifies the effects of fluoxetine and corticosterone on behavior, stress reactivity, and hippocampal neurogenesis. Neuropharmacology 105, 443–453. doi: 10.1016/j.neuropharm.2015.11.027, 26808316.

[ref55] Xiang X. Palasuberniam P. Pare R. (2024). Exploring the feasibility of estrogen replacement therapy as a treatment for perimenopausal depression: a comprehensive literature review. Med. (B. Aires.) 60:1076. doi: 10.3390/medicina60071076, 39064505. PMC11279181

